# Relationship Between Urinary 4-(Methylnitrosamino)-1-(3-Pyridyl)-1-Butanol and Lung Cancer Risk in the General Population: A Community-Based Prospective Cohort Study

**DOI:** 10.3389/fonc.2021.611674

**Published:** 2021-03-22

**Authors:** Eun Young Park, Min Kyung Lim, Eunjung Park, Jin-Kyoung Oh, Do-Hoon Lee

**Affiliations:** ^1^ Division of Cancer Prevention and Early Detection, National Cancer Control Institute, National Cancer Center, Goyang-si, South Korea; ^2^ Department of Cancer Control and Population Health, Graduate School of Cancer Science and Policy, National Cancer Center, Goyang-si, South Korea; ^3^ Department of Laboratory Medicine, National Cancer Center, Goyang-si, South Korea

**Keywords:** smoking, 4-(methylnitrosamino)-1-(3-pyridyl)-1-butanone (NNK), 4-(methylnitrosamino)-1-(3-pyridyl)-1-butanol (NNAL), lung cancer, biomarker

## Abstract

No published studies have prospectively evaluated the association between urinary 4-(methylnitrosamino)-1-(3-pyridyl)-1-butanol (NNAL) levels and lung cancer risk in the general population. Here, we conducted a prospective community-based cohort study in the Republic of Korea to evaluate the relationship between urinary NNAL levels and lung cancer risk using prediagnostic urine samples. This prospective cohort study was based on the Korean National Cancer Center Community Cohort. During the follow-up period, 173 primary lung cancer cases were identified. Total urinary NNAL levels were measured by liquid chromatography-tandem mass spectrometry, and data were analyzed using multivariable Cox proportional hazards regression models. The risk of lung cancer was significantly increased per unit of natural log-transformed urinary NNAL (HR, 1.27; 95% CI, 1.09–1.48), after adjusting for age, region, entry year into the cohort, education achievement, alcohol consumption status, BMI, smoking status, and urinary cotinine levels. Cox proportional-hazards models with NNAL quartiles also showed positive dose-response relationships with risk of lung cancer. A significantly increased risk of lung cancer was found in the fourth quartile of urinary NNAL levels (HR, 3.27; 95% CI, 1.37–7.79, P for trend < 0.01). After stratification with sex, the significant association remained in only men. Urinary NNAL levels are associated with the risk of lung cancer in the general population, and this association is independent from the quantification of cigarette smoking and nicotine uptake.

## Introduction

Lung cancer is the leading cause of cancer mortality and one of the most commonly diagnosed cancers in Korea and worldwide (1, 2). Although the carcinogenicity of occupational and environmental exposure of some factors such as radon, asbestos, silica, polycyclic aromatic hydrocarbons, and air pollution has been well identified for lung cancer (3), contribution of long-term cigarette smoking is an established causal factor for lung cancer and contributes to approximately 90% of lung cancer mortality (4).

Despite taking smoking history being the best validated method for assessing exposure and predicting lung cancer risk, it is difficult to be used for assessment of variation by smoking topography, is less reliable in assessing exposure reduction, and lacks accuracy to predict the internal dose or effects of smoking. Various biomarkers of tobacco smoke exposure have been proposed as useful methods for quantifying exposure to toxic constituents of smoke (5). Of these, cotinine and 4-(methylnitrosamino)-1-(3-pyridyl)-1-butanol (NNAL; metabolite of tobacco-specific nitrosamines [4-[methylnitrosamino]-1-[3-pyridyl]-1-butanone [NNK]) are highly specific and sensitive markers of tobacco exposure (6, 7). In particular, total urinary NNAL has an advantage over other smoking-related biomarkers (e.g., urinary cotinine) for epidemiological studies due to its relatively long half-life (i.e., 10 days to 3 weeks) (8, 9). Moreover, this measure is a sensitive and specific biomarker of exposure to secondhand smoke (10, 11).

Despite the introduction of precise and usable biological markers for tobacco smoke exposure, the association between urinary NNAL levels and lung cancer risk has only been evaluated in a limited number of nested case-control studies in China, Singapore, and the United States, most of which focused on men or a high-risk group of lung cancer such as current smokers (9, 12–15). To the best of our knowledge, no previous studies with prospective follow up design have evaluated the association between urinary NNAL levels and lung cancer risk in men and women in the general population.

Here, we evaluated the effect of urinary NNAL level on lung cancer risk in both men and women of a general population with a prospective community-based cohort in the Republic of Korea. Furthermore, the comparison and adjustment of lung cancer risk predicted by self-reported smoking history and urinary cotinine levels were considered to identify the independent and direct effect of urinary NNAL level on lung cancer risk.

## Materials and Methods

### Study Subjects

A total of 16,304 adult men and women aged more than 20 years who were included in the Korean National Cancer Center (KNCC) Community Cohort between 1993 and 2010 were considered to investigate known and potential environmental factors associated with cancer risk in the Republic of Korea. Details on the collection of baseline demographic, environmental, and lifestyle characteristics, anthropometric measurements, clinical laboratory tests, and biorepository samples were reported previously (16). Participants’ data were linked to the data from Korea Central Cancer Registry and National Statistics Korea for identification of new cancer cases and underwent follow-ups through 2016. All cancer cases newly developed in the cohort is identified through the data linkage without loss of follow-up. Primary lung cancer cases were defined as C33 or C34 according to the 10th edition of the International Classification of Diseases diagnostic codes.

**Figure 1:**
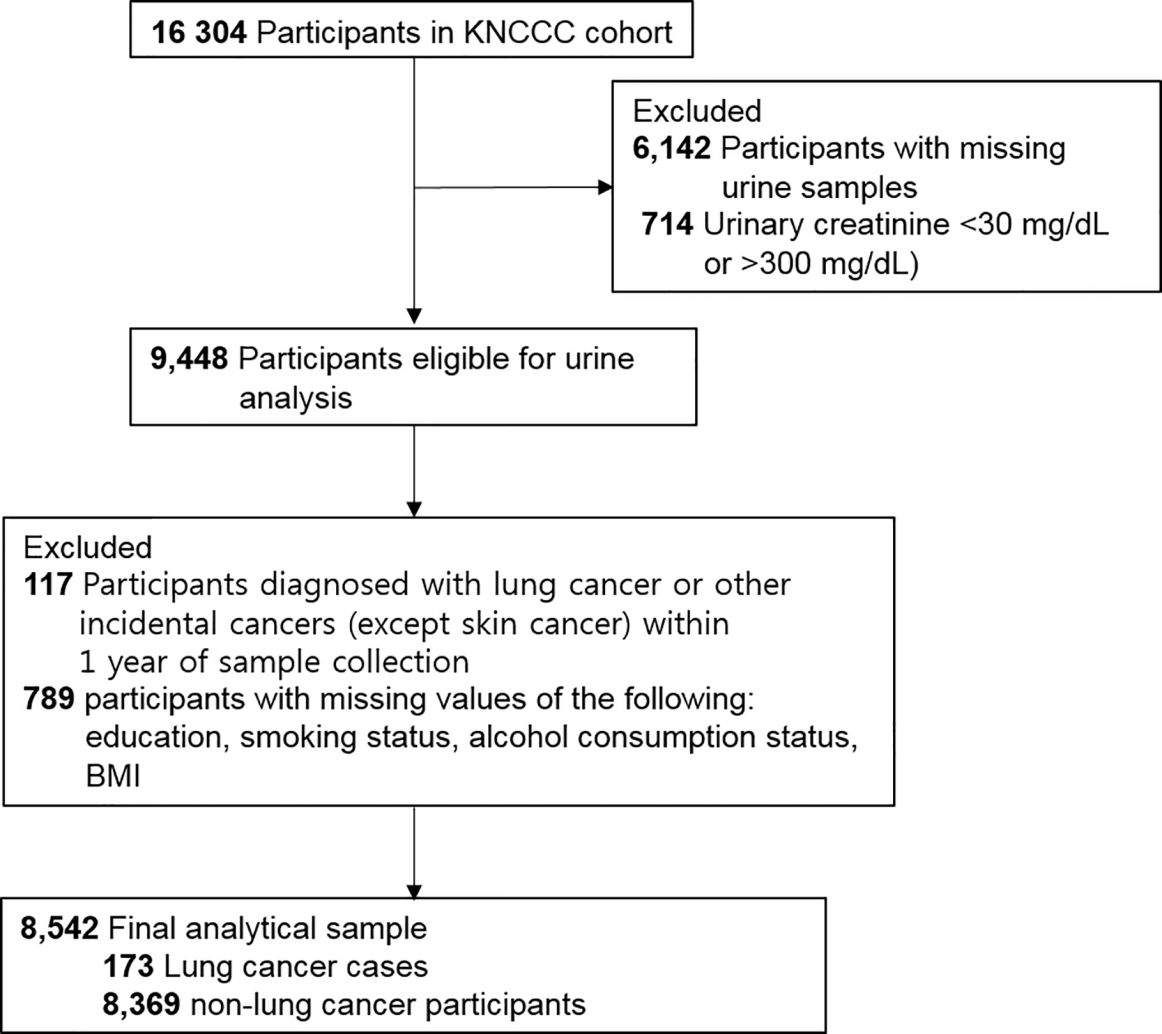
Flow diagram showing study sample derivation. KNCCC cohort, Korean National Cancer Center Community cohort; BMI, body mass index.

Of 16,304 participants, 9,448 men and women were eligible for urine analysis. We excluded participants who were diagnosed as having lung cancer and other incidental cancers within 1 year of cohort enrollment (n = 117), as well as those with missing data for covariates such as education achievement, smoking status, alcohol consumption status, and body mass index (BMI) (n = 789). As a result, we included 8,542 participants (173 lung cancer cases: men, 116; women, 57) in the final statistical analysis. A flow diagram detailing the recruitment and follow-up of study participants is presented in **Figure 1**.

KNCC institutional review board approved the study (no. NCC2017-0217), and all participants agreed with the research purpose and signed informed consent forms. The reporting of this cohort study followed the STROBE guidelines.

### Measures

Information on the following variables was adopted from the baseline survey: age, sex (men, women), region (Sancheong, Uiryeong, Chang-won, Chuncheon, Chungju, Haman), entry year into the cohort (from 1997 to 2010), education achievement (elementary school or less, middle school, high school or more), smoking status (nonsmoker, ever smoker with <30 pack-years, ever smoker with ≥30 pack-years), alcohol consumption status (never drinker, drinker with <24 g/day [12 g for women], drinker with ≥24 g/day), and obesity (BMI < 25 kg/m^2^, BMI ≥ 25 kg/m^2^).

The urinary cotinine and NNAL levels were measured by liquid chromatography-tandem mass spectrometry using modified methods that have been previously described (17, 18). The liquid chromatography unit used was an Agilent 1100 series (Agilent Technologies), and the tandem mass spectrometer was an API 4000 machine (AB SCIEX) equipped with an atmospheric pressure chemical ionization interface. The limits of detection (LOD) for cotinine and NNAL were 2 ng/ml and 0.25 pg/ml, respectively. 387 (4.52%, NNAL) and 2547 (29.81%, cotinine) of samples among this study participants had urinary concentration below the LOD. The creatinine levels in the urine were measured *via* colorimetry (Toshiba 2090 FR; Toshiba) and used to produce the creatinine-adjusted cotinine and NNAL concentration.

### Statistical Analyses

When the urinary cotinine and total NNAL levels were below the limit of quantification, half of them were assigned. Urinary cotinine and total NNAL levels were natural log (log_e_)-transformed to obtain the normal distribution and categorized into quartiles. This transformation yielded results in terms of easily interpretable hazard ratios (HRs) depending on the distribution of NNAL among non-lung cancer male participants. The data of non-lung cancer female participants were not suitable for assessing the dose-response of exposure to NNK because approximately 90% of women in this study were nonsmokers, and most women’s urinary total NNAL levels were very low (**Table 2**).

Censoring was done at the time of death, or a diagnosis of any other cancer, whichever came first or end of follow-up (December 31, 2016).

We examined differences of baseline characteristics such as age, sex, regions, year of entry into the cohort, education achievement, smoking status, alcohol consumption status, and obesity between lung cancer cases and non-lung cancer participants.

There was no violation of proportional hazards assumption, when Schoenfeld residuals were evaluated to assess the validity of the assumptions. Associations between lung cancer risk and smoking status (pack-years), urinary cotinine levels, and NNAL levels were assessed to estimate HRs and 95% CIs using multivariable Cox proportional hazards regression models, applying age as the time scale (19). First, two separate models were evaluated: the first model was unadjusted; the second model was adjusted for age (continuous variable), sex (only overall dataset), entry year into the cohort, region, education achievement, alcohol consumption status, and BMI (continuous variable).

From these analyses, three additional statistical models were conducted to evaluate the sole effect of urinary NNAL levels on lung cancer risk. The first model was further adjusted for smoking status (pack-years and continuous variable). The second model was further adjusted for urinary cotinine levels (log_e_ and continuous variable) instead of smoking status. The third model was further adjusted for both smoking status (pack-years and continuous variable), and urinary cotinine levels (log_e_ and continuous variable). Additionally, time-lag analyses, which censor cases occurring between baseline and 2 and 5 years after entry, were performed.

Sensitivity analysis has been done with the multiple imputation on data from missing covariates (n = 789), considering limited power of the study (20, 21).

We also investigated the possibility of nonlinear associations such as an inverted U-shaped association using restricted cubic spline functions.

Statistical analysis has been done with statistical package of SAS version 9.4 (SAS Institute, Cary, NC, USA), and all tests were two-sided with a significance level set at a P value less than 0.05.

## Results

**Table 1** shows baseline characteristics of the study participants. The median of follow-up period was 12.50 years (interquartile range: 8.75–16.25, 939852.65 person-years at risk). Of the 173 lung cancer cases, in men, the cases were 113 and incidence rate per 100 000 person years was 332.39. On the other hand, in women, the cases were 57 and incidence rate per 100 000 person years was 96.47. Urinary total NNAL and cotinine levels were much higher in men than women (i.e., total NNAL (GM ± GSD, pg/mg creatinine): 20.9 ± 9.76 vs. 4.51 ± 6.30, total cotinine (GM ± GSD, ng/mg creatinine): 36.49 ± 28.28 vs. 2.95 ± 7.55). Most of women were non-smokers (92.01%). In contrast, most of men were former- or current smokers (80.24%).

**Table 1:** Baseline characteristics of study participants.

	Men (N = 3,351)	Women (N = 5,191)
**Cases of lung cancer, n (%)**	116 (3.46)	57 (1.10)
**Incidence rate per 100 000 person years**	332.39	96.47
**NNAL, GM ± GSD**	20.9 ± 9.76	4.51 ± 6.30
**Cotinine, GM ± GSD**	36.49 ± 28.28	2.95 ± 7.55
Smoking status, pack-years, mean ± SD^a^	32.41 ± 20.82	12.99 ± 12.25
Non-smoker	662 (19.76)	4776 (92.01)
<30 pack-years	1346 (40.17)	373 (7.19)
≥30 pack-years	1343 (40.08)	42 (0.81)
**Age, years, mean ± SD**	59.53 ± 10.77	59.46 ± 10.88
20-29	10 (0.30)	9 (0.17)
30-39	115 (3.43)	230 (4.43)
40-49	505 (15.07)	804 (15.49)
50-59	898 (26.80)	1258 (24.23)
60-69	1227 (36.62)	1991 (38.35)
70+	596 (17.79)	899 (17.32)
**Region, n (%)**		
San-cheong	1326 (39.57)	2151 (41.44)
Ui-ryeong	113 (3.37)	208 (4.01)
Chang-won	493 (14.71)	794 (15.30)
Choon-cheon	45 (1.34)	63 (1.21)
Choong-joo	309 (9.22)	472 (9.09)
Ham-an	1065 (31.78)	1503 (28.95)
**Year of cohort entry, n (%)**		
1997	326 (9.73)	421 (8.11)
1999	168 (5.01)	271 (5.22)
2000	164 (4.89)	193 (3.72)
2001	276 (8.24)	484 (9.32)
2002	92 (2.75)	115 (2.22)
2003	252 (7.52)	367 (7.07)
2004	579 (17.28)	917 (17.67)
2005	308 (9.19)	488 (9.40)
2006	419 (12.50)	630 (12.14)
2007	237 (7.07)	403 (7.76)
2008	260 (7.76)	362 (6.97)
2009	141 (4.21)	263 (5.07)
2010	129 (3.85)	277 (5.34)
**Education achievement, n (%)**		
None	375 (11.19)	1685 (32.46)
Middle school	2080 (62.07)	2977 (57.35)
High school or more	896 (26.74)	529 (10.19)
**Alcohol consumption status, n (%)**		
Never	848 (25.31)	4127 (79.50)
< 24 g per day (12g for women)	1325 (39.54)	866 (16.68)
≥24 g per day	1178 (35.15)	198 (3.81)
**Obesity (BMI), mean ± SD**	23.28 ± 2.90	23.98 ± 3.31
No (BMI<25 kg/m^2^), n (%)	2447 (73.02)	3351 (64.55)
Yes (BMI≥25 kg/m^2^), n (%)	904 (26.98)	1840 (35.45)

a except nonsmokers.

GM, Geometric means; GSD, Geometry standard deviation; BMI, body mass index.

The average age of lung cancer cases was 65 and older than the non-lung cancer participants. They had higher proportions of men and cigarette smokers compared with the non-lung cancer participants (**Table S1**). Distributions of smoking status (pack-years), urinary cotinine levels, and total NNAL levels are presented in **Table 2**. All measures were significantly higher in the lung cancer cases than in the non-lung cancer participants (*P* < 0.01) for both sexes, except for pack-years in women, which was not significantly higher in the lung cancer cases than in the non-lung cancer participants. Creatinine-adjusted urinary levels of cotinine and total NNAL were highly correlated (Spearman ρs: men, 0.86; women, 0.65; *Ps* < 0.01) (data not shown).

**Table 2:** Distribution of smoking status and urine cotinine and NNAL concentrations stratified by lung cancer cases and non-lung cancer participants.

	Total	Lung cancer cases (n = 173)								Non-lung cancer participants (n =8369)								*P*
	<LOD (%)	n	<LOD (%)	GM (95% CI)	Distribution (quartiles)					n	<LOD (%)	GM (95% CI)	Distribution (quartiles)					
					Min	Q1	Median	Q3	Max				Min	Q1	Median	Q3	Max	
**Overall**																		
Smoking status, pack-years		128		29.72 (25.75, 34.31)	0.75	21.15	34.50	50.00	138.00	2976		21.42 (20.70, 22.17)	0.05	14.50	26.00	40.00	200.00	<0.01
Non-smoker		45		N/A	N/A	N/A	N/A	N/A	N/A	5393		N/A	N/A	N/A	N/A	N/A	N/A	N/A
<30 pack-years		51		14.22 (11.41, 17.73)	0.75	11.50	19.50	24.00	29.00	1668		12.03 (11.52, 12.55)	0.05	8.55	16.05	22.50	29.75	0.18
≥30 pack-years		77		48.43 (44.86, 52.28)	30.00	40.00	47.00	56.00	138.00	1308		44.72 (43.97, 45.49)	30.00	35.25	42.00	52.00	200.00	0.03
NNAL	4.52	173	1.16	51.52 (37.09, 71.59)	0.10	8.46	150.14	282.79	2121.57	8369	4.59	7.93 (7.57, 8.30)	0.02	2.03	5.99	32.90	38071.90	<0.01
Cotinine	29.81	173	12.72	96.74 (59.42, 157.50)	0.25	2.90	728.92	1593.07	6374.50	8369	30.16	7.52 (7.07, 8.00)	0.17	0.92	2.56	21.91	95796.00	<0.01
**Men**																		
Smoking status, pack-years		110		34.41 (30.38, 38.97)	2.00	24.00	41.00	53.00	138.00	2579		25.20 (24.45, 25.98)	0.30	18.00	29.00	42.00	200.00	<0.01
Non-smoker		6		N/A	N/A	N/A	N/A	N/A	N/A	656		N/A	N/A	N/A	N/A	N/A	N/A	N/A
<30 pack-years		38		17.28 (14.35, 20.79)	2.00	15.00	20.50	24.00	29.00	1308		14.40 (13.86, 14.96)	0.30	11.00	18.00	24.00	29.75	0.11
≥30 pack-years		72		49.50 (45.74, 53.57)	30.00	41.00	47.50	56.85	138.00	1271		44.85 (44.09, 45.63)	30.00	36.00	42.00	52.50	200.00	<0.01
NNAL	2.27	116	0.00	102.30 (73.38, 142.50)	0.63	47.19	195.87	327.97	2054.73	3235	2.35	19.79 (18.30, 21.41)	0.02	3.21	19.30	163.89	38071.90	<0.01
Cotinine	17.67	116	6.03	281.30 (168.80, 468.60)	0.25	148.07	990.38	1785.45	6374.50	3235	18.08	33.92 (30.23, 38.05)	0.18	1.41	13.83	1082.84	95796.00	<0.01
**Women**																		
Smoking status, pack-years		18		12.15 (6.95, 21.23)	0.75	5.50	13.85	30.00	45.00	397		7.44 (6.59, 8.40)	0.05	4.05	8.75	18.50	76.50	0.10
Non-smoker		39		N/A	N/A	N/A	N/A	N/A	N/A	4737		N/A	N/A	N/A	N/A	N/A	N/A	N/A
<30 pack-years		13		8.05 (4.25, 15.25)	0.75	4.00	11.00	14.70	27.50	360		6.25 (5.55, 7.05)	0.05	3.68	7.80	15.00	29.00	0.44
≥30 pack-years		5		35.38 (28.99, 43.19)	30.00	31.80	34.00	38.00	45.00	37		40.47 (37.38, 43.80)	30.00	33.00	38.40	46.00	76.50	0.23
NNAL	5.97	57	3.51	12.77 (7.01, 23.26)	0.10	3.09	8.47	105.03	2121.57	5134	6.00	4.46 (4.24, 4.69)	0.02	1.64	4.30	12.30	25561.43	<0.01
Cotinine	37.64	57	26.32	11.02 (4.88, 24.89)	0.40	1.23	2.56	308.61	3875.00	5134	37.77	2.91 (2.76, 3.08)	0.17	0.81	1.69	4.98	6069.52	<0.01

The associations of smoking status categorized by pack-years and creatinine-adjusted urinary cotinine levels on incident lung cancer risk are presented in **Table 3**. After adjusting for age, region, entry year into the cohort, education achievement, alcohol consumption status, and BMI, the following variables were significantly associated with increase of lung cancer risk in the Cox proportional hazards models with a dose-response relationship (P for trend <0.01): smoking intensity (men, <30 pack-years: HR, 3.49; 95% CI, 1.46–8.33, ≥30 pack-years: HR, 5.17; 2.23–11.98; women, <30 pack-years: HR, 3.26; 95% CI, 1.67–6.35, ≥30 pack-years: HR, 10.45; 95% CI, 3.85–28.39), urinary cotinine level (men, third quartile: HR, 3.77; 95% CI, 1.86–7.67, fourth quartile: HR, 5.53; 95% CI, 2.73–11.18; women, fourth quartile: HR, 3.12; 95% CI, 1.38–7.09), urinary NNAL level (men, third quartile: HR, 3.30; 95% CI, 1.53–7.09, fourth quartile: HR, 7.69; 95% CI, 3.69–16.02; women, fourth quartile: HR, 4.68; 95% CI, 2.03–10.77). The results from the continuous models were similar (**Table 3**).

**Table 3:** Association between smoking status, urine cotinine and NNAL concentrations, and incident lung cancer risk.

		Overall (N = 8542)			Men (N = 3351)			Women (N = 5191)		
		Cases (%)	HR (95% C.I.)	P for trend	Cases (%)	HR (95% C.I.)	P for trend	Cases (%)	HR (95% C.I.)	P for trend
Model 1										
Smoking status	Continuous (log_e_)		1.02 (1.02, 1.03)			1.02 (1.01, 1.02)			1.05 (1.03, 1.08)	
	Non-smoker	45 (0.83)	Reference	<0.01	6 (0.91)	Reference	<0.01	39 (0.82)	Reference	<0.01
	<30 pack-years	51 (2.97)	4.05 (2.71, 6.05)		38 (2.82)	3.63 (1.53, 8.59)		13 (3.49)	3.72 (1.96, 7.06)	
	≥30 pack-years	77 (5.56)	6.77 (4.68, 9.79)		72 (5.36)	5.39 (2.34, 12.40)		5 (11.90)	10.87 (4.20, 28.14)	
Cotinine	Continuous (log_e_)		1.30 (1.24, 1.37)			1.26 (1.18, 1.35)			1.21 (1.11, 1.33)	
(ng/mg creatinine)	< 1.41	28 (0.92)	Reference	<0.01	10 (1.25)	Reference	<0.01	18 (0.81)	Reference	<0.01
	1.41–13.38	35 (1.16)	1.21 (0.74, 2.00)		13 (1.61)	1.31 (0.57, 2.99)		22 (0.99)	1.17 (0.63, 2.18)	
	13.38–1082.84	45 (3.28)	3.61 (2.25, 5.79)		38 (4.52)	3.90 (1.94, 7.84)		7 (1.32)	1.47 (0.61, 3.51)	
	≥ 1082.84	65 (5.84)	6.91 (4.43, 10.77)		55 (6.09)	5.81 (2.95, 11.46)		10 (4.76)	4.75 (2.19, 10.32)	
NNAL	Continuous (log_e_)		1.52 (1.41, 1.64)			1.51 (1.36, 1.68)			1.29 (1.13, 1.47)	
(pg/mg creatinine)	< 3.21	25 (0.84)	Reference	<0.01	9 (1.11)	Reference	<0.01	16 (0.74)	Reference	<0.01
	3.21–19.30	31 (1.10)	1.23 (0.72, 2.08)		11 (1.39)	1.27 (0.53, 3.07)		20 (0.99)	1.22 (0.63, 2.36)	
	19.30–163.89	36 (2.25)	2.55 (1.53, 4.25)		28 (3.38)	3.18 (1.50, 6.75)		8 (1.03)	1.20 (0.51, 2.81)	
	≥ 163.89	81 (6.99)	8.47 (5.41, 13.27)		68 (7.42)	7.41 (3.69, 14.89)		13 (5.39)	5.74 (2.75, 11.97)	
Model 2										
Smoking status	Continuous (log_e_)		1.02 (1.01, 1.02)			1.02 (1.01, 1.02)			1.06 (1.03, 1.08)	
	Non-smoker	45 (0.83)	Reference	<0.01	6 (0.91)	Reference	<0.01	39 (0.82)	Reference	<0.01
	<30 pack-years	51 (2.97)	3.47 (2.12, 5.70)		38 (2.82)	3.49 (1.46, 8.33)		13 (3.49)	3.26 (1.67, 6.35)	
	≥30 pack-years	77 (5.56)	5.71 (3.31, 9.85)		72 (5.36)	5.17 (2.23, 11.98)		5 (11.90)	10.45 (3.85, 28.39)	
Cotinine	Continuous (log_e_)		1.21 (1.15, 1.28)			1.25 (1.17, 1.35)			1.16 (1.05, 1.28)	
(ng/mg creatinine)	< 1.41	28 (0.92)	Reference	<0.01	10 (1.25)	Reference	<0.01	18 (0.81)	Reference	0.03
	1.41–13.38	35 (1.16)	1.18 (0.71, 1.96)		13 (1.61)	1.32 (0.57, 3.04)		22 (0.99)	0.99 (0.52, 1.88)	
	13.38–1082.84	45 (3.28)	2.55 (1.55, 4.19)		38 (4.52)	3.77 (1.86, 7.67)		7 (1.32)	1.08 (0.44, 2.66)	
	≥ 1082.84	65 (5.84)	3.93 (2.40, 6.44)		55 (6.09)	5.53 (2.73, 11.18)		10 (4.76)	3.12 (1.38, 7.09)	
NNAL	Continuous (log_e_)		1.38 (1.27, 1.51)			1.49 (1.34, 1.66)			1.25 (1.08, 1.44)	
(pg/mg creatinine)	< 3.21	25 (0.84)	Reference	<0.01	9 (1.11)	Reference	<0.01	16 (0.74)	Reference	<0.01
	3.21–19.30	31 (1.10)	1.21 (0.71, 2.07)		11 (1.39)	1.34 (0.55, 3.27)		20 (0.99)	1.14 (0.57, 2.28)	
	19.30–163.89	36 (2.25)	2.10 (1.23, 3.58)		28 (3.38)	3.30 (1.53, 7.09)		8 (1.03)	1.07 (0.44, 2.63)	
	≥ 163.89	81 (6.99)	5.40 (3.25, 8.97)		68 (7.42)	7.69 (3.69, 16.02)		13 (5.39)	4.68 (2.03, 10.77)	

Model 1, unadjusted estimates; Model 2, Adjusted for age, sex, area, enrollment year, alcohol consumption, BMI (kg/m2) and education achievement.

**Table 4** presents specific associations between urinary NNAL levels and lung cancer risk. The risk of lung cancer was significantly increased per unit of NNAL (HR, 1.33; 95% CI, 1.22–1.45), after adjusting for age, region, entry year into the cohort, education achievement, alcohol consumption status, BMI, and smoking status (pack-year). When stratified with sex, the HR for men was 1.44 (95% CI, 1.29–1.61) but the HR for women was 1.12 (95% CI, 0.96–1.31). After additional adjustment for urinary cotinine levels, the associations were consistent (men: HR, 1.40; 95% CI, 1.16–1.69; women: HR, 1.12; 95% CI, 0.89–1.41). Furthermore, significant nonlinear associations between urinary NNAL levels and lung cancer risk were found for the overall cohort (P = 0.03), as well as for men (P = 0.03) but not for women (P = 0.65) (**Figure S1**).

**Table 4:** Association between urine NNAL concentrations and incident lung cancer risk.

NNAL (pg/mg creatinine)	Overall (N = 8542)			Men (N = 3351)			Women (N = 5191)		
	Cases (%)	HR (95% C.I.)	P for trend	Cases (%)	HR (95% C.I.)	P for trend	Cases (%)	HR (95% C.I.)	P for trend
Model 1									
Continuous (log_e_)		1.33 (1.22, 1.45)			1.44 (1.29, 1.61)			1.12 (0.96, 1.31)	
< 3.21	25 (0.84)	Reference	<0.01	9 (1.11)	Reference	<0.01	16 (0.74)	Reference	0.13
3.21–19.30	31 (1.10)	1.19 (0.69, 2.04)		11 (1.39)	1.32 (0.54, 3.22)		20 (0.99)	1.11 (0.55, 2.23)	
19.30–163.89	36 (2.25)	2.03 (1.19, 3.46)		28 (3.38)	3.20 (1.49, 6.87)		8 (1.03)	0.94 (0.38, 2.32)	
≥ 163.89	81 (6.99)	4.45 (2.66, 7.45)		68 (7.42)	6.42 (3.06, 13.49)		13 (5.39)	2.64 (1.02, 6.85)	
Model 2									
Continuous (log_e_)		1.30 (1.12, 1.51)			1.42 (1.18, 1.71)			1.15 (0.91, 1.45)	
< 3.21	25 (0.84)	Reference	<0.01	9 (1.11)	Reference	<0.01	16 (0.74)	Reference	0.26
3.21–19.30	31 (1.10)	1.15 (0.67, 1.99)		11 (1.39)	1.27 (0.52, 3.12)		20 (0.99)	1.15 (0.57, 2.34)	
19.30–163.89	36 (2.25)	1.63 (0.81, 3.27)		28 (3.38)	2.43 (0.88, 6.69)		8 (1.03)	1.13 (0.40, 3.22)	
≥ 163.89	81 (6.99)	3.59 (1.51, 8.54)		68 (7.42)	5.01 (1.55, 16.15)		13 (5.39)	5.37 (1.10, 26.16)	
Model 3									
Continuous (log_e_)		1.27 (1.09, 1.48)			1.40 (1.16, 1.69)			1.12 (0.89, 1.41)	
< 3.21	25 (0.84)	Reference	<0.01	9 (1.11)	Reference	<0.01	16 (0.74)	Reference	0.33
3.21–19.30	31 (1.10)	1.15 (0.67, 1.98)		11 (1.39)	1.27 (0.52, 3.12)			1.16 (0.57, 2.37)	
19.30–163.89	36 (2.25)	1.67 (0.83, 3.36)		28 (3.38)	2.54 (0.92, 6.98)			1.16 (0.40, 3.38)	
≥ 163.89	81 (6.99)	3.27 (1.37, 7.79)		68 (7.42)	4.67 (1.46, 14.99)			4.25 (0.82, 21.99)	

Model 1 is adjusted for age, sex, area, enrollment year, alcohol consumption, BMI (kg/m^2^), education achievement and smoking status (pack-year).

Model 2 is adjusted for age, sex, area, enrollment year, alcohol consumption, BMI (kg/m^2^), education achievement and cotinine level.

Model 3 is adjusted for age, age, sex, area, enrollment year, alcohol consumption, BMI (kg/m^2^), education achievement, smoking status (pack-year) and cotinine level.

As shown in analysis results with Cox proportional-hazards models, NNAL levels in continuous variable and in quartile showed positive dose-response relationships with risk of lung cancer. A significantly increased risk of lung cancer was found in the fourth quartile of urinary NNAL levels in all models, including the full adjustment model (HR, 3.27; 95% CI, 1.37–7.79, P for trend <0.01). After stratification with sex, the significant association remained in only men (men: HR, 4.67; 95% CI, 1.46–14.99, P for trend <0.01, women: HR, 4.25; 95% CI, 0.82–21.99, P for trend =0.33) (**Table 4**).

The results from sensitivity analysis with the multiple imputation are similar with the results from primary analysis (**Tables S2** and **S3**). The findings from time-lag analyses which censor cases occurring between baseline and 2 and 5 years after entry were also not much different from above (**Tables S4–S7**).

## Discussion

To the best of our knowledge, this is the first community-based prospective cohort study on associations between urinary NNAL levels and lung cancer risk in both men and women. Our results suggest that the risk of lung cancer increased based on the level of urinary NNAL increase in all models, with consistent associations in both men and women. Furthermore, comprehensive adjustment for potential confounders including smoking status, alcohol consumption, obesity, and urinary cotinine levels as a biomarker of exposure to active or secondhand smoking strengthened the meaning of the results.

These findings on associations between urinary total NNAL and lung cancer risk are consistent with previous studies for smokers. In the United States-based case-control study nested within the Prostate, Lung, Colorectal, and Ovarian Cancer Screening Trial, lung cancer risk per standard deviation (SD) of total serum NNAL levels increased 1.57 folds (P = 0.018) after multivariate adjustment including number of years of smoking, serum cotinine, and phenanthrene tetraol (15). In the nested case-control study of lung cancer within the Shanghai Cohort, total urinary NNAL was associated with lung cancer risk (odds ratio, 2.04; 95% CI, 1.02–4.05) in people with the highest tertile (≥0.210 pmol/mg creatinine [43.94 pg/mg creatinine]) compared with those in lowest tertile (≤0.105 pmol/mg creatinine [21.97 pg/mg creatinine]), after adjustment for total urinary cotinine levels and smoking intensity and duration (9). Similarly, a case-control study nested within the Singapore Chinese Health Study reported an odds ratio for lung cancer of 2.64 (95% CI, 1.10–6.34) in smokers in the highest tertile (≥0.820 pmol/mg creatinine [171.577 pg/mg creatinine]) relative to those in the lowest tertile (≤0.468 pmol/mg creatinine [97.924 pg/mg creatinine]) of urinary total NNAL levels, after multivariate adjustment including urinary total cotinine levels (13, 14). However, the lung cancer risk associated with NNAL in this study was estimated to be relatively higher (men: HR, 4.67; 95% CI, 1.46–14.99; women: HR, 4.25; 95% CI, 0.82–21.99 in the highest quartile with ≥163.89 pg/mg creatinine compared to the lowest quartile with <3.22 pg/mg creatinine) than those from other previous studies (9, 13, 14), as approximately half of the study participants were self-reported nonsmokers, who have lower level of NNAL than smokers who used as reference group of other previous studies, and they were mostly categorized as a reference group in the present study. Additionally, the strength of the associations may be attenuated in our cohort study. Without further adjustment for pack-years and urinary cotinine levels, the risk of lung cancer in men increased from the third quartile (19.30–163.89 pg/mg creatinine: HR, 3.30; 95% CI, 1.53–7.09) to the fourth quartile (≥163.89 pg/mg creatinine: HR, 7.69; 95% CI, 3.69–16.02) in the model. These HRs were similar to those from the model that further included pack-years to adjust for the accumulated effect of smoking before entering the cohort (third quartile: HR, 3.20; 95% CI, 1.49–6.87; fourth quartile: HR, 6.42; 95% CI, 3.06–13.49). After further adjustment for pack-years and urinary cotinine levels (as a proxy for current exposure to smoking, including secondhand smoking), the risk of lung cancer disappeared in the third quartile (HR, 2.54; 95% CI, 0.92–6.98) and was much lower in the fourth quartile (HR, 4.67; 95% CI, 1.46–14.99). The adjustment for urinary cotinine levels has the potential to underestimate the actual carcinogenic effects of NNK as well as overall exposure level of tobacco smoke, which does not occur without tobacco smoking exposure, because most of the absorbed nicotine from tobacco smoke exposure is metabolized as cotinine. On the other hand, the risk of lung cancer in the model with adjustment for both pack-years of smoking and urinary cotinine levels may not be the sole effect of NNK, as other carcinogens from tobacco exposure may contribute to the risk increase. Thus, further epidemiologic analyses are needed to evaluate the effect of multiple exposure of numerous carcinogens caused by smoking in the future.

The results of the current study also suggest that tobacco-related biomarkers such as cotinine and NNAL may better assess the health effects of smoke exposure. Biomarkers may provide a direct method to assess exposure to carcinogens and toxicants in individuals, as well as to reduce the misclassification and imprecision introduced by self-reported smoking history (10, 22). For this reason, Hecht et al. proposed the application of tobacco carcinogen and toxicant biomarkers to identify individuals at high risk for cancer (8). However, biomarker measures have some limitations to estimate internal dose, which shows considerable interindividual variability in the rate of elimination caused by a number of factors such as physiological factors (e.g., sex, age, and diet), genetic factors (e.g., genetic variation in CYP2A6 and UDP-glucuronosyltransferase (UGT), medications, smoking itself, and others (7, 23). Additionally, total NNAL does not entirely reflect metabolic activation of NNK. That is, increased metabolic activation of NNK (e.g., NNK DNA adducts) would increase cancer risk but would decrease total NNAL levels. Furthermore, total NNAL accounts for only 12–17% of the NNK dose (24). This means that total NNAL as a biomarker may not fully reflect the effects of internal NNK on cancer, and the actual effect of NNK exposure from smoking, including secondhand smoke on cancer, may be greater than those seen in the current study.

The International Agency for Research on Cancer (IARC) evaluated NNK and N’-nitrosonornicotine (NNN) and found them to be carcinogenic to humans (i.e., group 1) due to sufficient evidence from animals and strong mechanistic evidence in humans (4). NNK and NNN are products of nitrosation of nicotine and other tobacco alkaloids. Substantial quantities of them are formed during tobacco curing and processing, as well as during smoking (4, 25). Many studies in animals have found that different routes of exposure to NNK induce benign and malignant tumors in the lungs, nasal cavity, trachea, pancreas, and liver (4, 8). NNK induces formation of DNA adducts, considered as tumor initiation (i.e., evasion of the repair system), and miscoding during DNA replication, which then results in deleterious mutations in oncogenes and tumor suppressor genes. Additionally, NNK binds to nicotinic acetylcholine receptors, promoting tumor growth by deregulating cell proliferation, survival, migration, and invasion. Furthermore, NNK, along with NNN, synergistically causes cancer (25).

Recently, tobacco companies have promoted novel tobacco products that purport to reduce carcinogen exposure, but risk reduction and its relation to exposure are not simple to estimate because tobacco using behaviors and response to the carcinogens may be different based on the tobacco user and tobacco product (26). Therefore, biomarker measurements would be critical for rapid evaluation of these novel tobacco products. Particularly, nicotine-related metabolites such as NNAL may be useful because they are specific to tobacco, including novel tobacco products. Additionally, nicotine levels in the aerosols of novel products are not much different from those in the smoke of the conventional cigarette, despite the claim from tobacco companies that other carcinogens and toxicants are reduced in the novel products (27).

There are several limitations in this study. First, one-spot urine samples were collected at the baseline recruitment. We could not ascertain changes of exposure to NNK after entering the cohort. However, these changes would occur in both lung cancer cases and non-lung cancer participants and may lead to underestimation of the real effects. The timing of obtaining urine samples could also affect the concentration of the urinary biomarkers measured. However, spot urine was taken after at least 6 h of overnight fasting in the entire cohort. Additionally, a single measurement of urinary NNAL level is generally accepted as an appropriate proxy for exposure to smoking, as shown in previous studies (9, 28). Second, lifestyle changes, including those in smoking history (e.g., intensity of cigarette smoking, types of smoked tobacco, or change of preferred tobacco products), could not be considered during the follow up period. Third, the effect of potential confounding factors might be involved in the results of present study, as with most observational studies. Although adjustment has been done for age, sex, region, education, smoking history, alcohol consumption, and obesity to minimize the limitation, other confounders unobserved or bias due to self-reporting may still influence on the results. Finally, relatively small sample size and small number of case had limited statistical power of the analysis in the present study, in particular, in women who has very low smoking prevalence and relatively small number of lung cancer case identified then risk estimates had wide confidence intervals. Thus, further studies with larger sample sizes and longer follow up to see enough number of lung cancer cases are needed to validate our findings, especially for women.

In conclusion, urinary NNAL levels are associated with the risk of lung cancer in the general population. This association is independent from the quantification of cigarette smoking and nicotine uptake. These results suggest that public health actions for tobacco product regulation should be taken and comprehensive information on tobacco carcinogens should be provided to the public.

## Data Availability Statement

The raw data supporting the conclusions of this article will be made available by the authors, without undue reservation.

## Ethics Statement

The studies involving human participants were reviewed and approved by the KNCC institutional review board (no. NCC2017-0217) and informed consent forms were signed by all participants. The patients/participants provided their written informed consent to participate in this study.

## Author Contributions

ML: conceptualization, methodology, writing (review and editing), and supervision. EYP: methodology and writing (original draft preparation). EP: statistical analysis and visualization. J-KO: investigation and writing (review and editing). D-HL: measurement of urinary cotinine and NNAL levels and writing (review and editing). All authors contributed to the article and approved the submitted version.

## Funding

This work was supported by the National Cancer Center (grant number: NCC-1315101), Ministry of Food and Drug Safety (grant number: 14182MFDS977), and the National Research Foundation of Korea (grant number: 2020R1A2C201229511). The funders had no role in the design or conduct of the study, the collection, management, analysis, and interpretation of the data, the preparation, review, or approval of the manuscript, or the decision to submit the manuscript for publication.

## Conflict of Interest

The authors declare that the research was conducted in the absence of any commercial or financial relationships that could be construed as a potential conflict of interest.

## Abbreviations

BMI, body mass index; HR, hazard ratio; IARC, International Agency for Research on Cancer; KNCC, Korean National Cancer Center; NNAL, 4-(methylnitrosamino)-1-(3-pyridyl)-1-butanol; NNK, 4-(methylnitrosamino)-1-(3-pyridyl)-1-butanone; NNN, N’-nitrosonornicotine.
