# Supplementary material for: Relationship Between Urinary 4-(Methylnitrosamino)-1-(3-Pyridyl)-1-Butanol and Lung Cancer Risk in the General Population: A Community-Based Prospective Cohort Study
Source: Front Oncol. 2021 Mar 22;11:611674. doi: 10.3389/fonc.2021.611674 (PMC8019966; doi:10.3389/fonc.2021.611674)
Supplement: Supplementary file 1 [file DataSheet_1.docx]

Table S1. Baseline characteristics of lung cancer cases comparing with non-lung cancer participants

|  | **Lung cancer cases (n=173)** | **Non-lung cancer participants (n=8369)** | **P-value** |
| --- | --- | --- | --- |
| **Age, years, mean±SD** | 64.53 ± 8.02 | 59.38 **±** 10.86 | <0.01 |
| 20-29 | 0 (0.00) | 19 (0.23) | <0.01 |
| 30-39 | 1 (0.58) | 344 (4.11) |  |
| 40-49 | 7 (4.05) | 1302 (15.56) |  |
| 50-59 | 31 (17.92) | 2125 (25.39) |  |
| 60-69 | 85 (49.13) | 3133 (37.44) |  |
| 70+ | 49 (28.32) | 1446 (17.28) |  |
| **Sex, n (%)** |  |  |  |
| Men | 116 (67.05) | 3235 (38.65) | <0.01 |
| Women | 57 (32.95) | 5134 (61.35) |  |
| **Region, n (%)** |  |  |  |
| San-cheong | 45 (26.01) | 3432 (41.01) | <0.01 |
| Ui-ryeong | 8 (4.62) | 313 (3.74) |  |
| Chang-won | 19 (10.98) | 1268 (15.15) |  |
| Choon-cheon | 4 (2.31) | 104 (1.24) |  |
| Choong-joo | 16 (9.25) | 765 (9.14) |  |
| Ham-an | 81 (46.82) | 2487 (29.72) |  |
| **Year of cohort entry, n (%)** |  |  |  |
| 1997 | 25 (14.45) | 722 (8.63) | <0.01 |
| 1999 | 16 (9.25) | 423 (5.05) |  |
| 2000 | 11 (6.36) | 346 (4.13) |  |
| 2001 | 20 (11.56) | 740 (8.84) |  |
| 2002 | 4 (2.31) | 203 (2.43) |  |
| 2003 | 22 (12.72) | 597 (7.13) |  |
| 2004 | 25 (14.45) | 1471 (17.58) |  |
| 2005 | 9 (5.20) | 787 (9.40) |  |
| 2006 | 16 (9.25) | 1033 (12.34) |  |
| 2007 | 11 (6.36) | 629 (7.52) |  |
| 2008 | 10 (5.78) | 612 (7.31) |  |
| 2009 | 2 (1.16) | 402 (4.80) |  |
| 2010 | 2 (1.16) | 404 (4.83) |  |
| **Education achievement, n (%)** |  |  |  |
| None | 49 (28.32) | 2011 (24.03) | 0.09 |
| Middle school | 105 (60.69) | 4952 (59.17) |  |
| High school or more | 19 (10.98) | 1406 (16.80) |  |
| **Smoking status, pack-years, mean±SD^a^** | 37.90 **±** 23.51 | 29.46 **±** 20.77 | <0.01 |
| Non-smoker | 45 (26.01) | 5393 (64.44) | <0.01 |
| <30 pack-years | 51 (29.48) | 1668 (19.93) |  |
| ≥30 pack-years | 77 (44.51) | 1308 (15.63) |  |
| **Alcohol consumption status, n (%)** |  |  |  |
| Never | 78 (45.09) | 4897 (58.51) | <0.01 |
| < 24 g per day (12g for women) | 55 (31.79) | 2136 (25.52) |  |
| $\geq$24 g per day | 40 (23.12) | 1336 (15.96) |  |
| **Obesity (BMI), mean±SD** | 22.40 **±** 2.87 | 23.73 **±** 3.18 | <0.01 |
| No (BMI<25 kg/m^2^), n (%) | 144 (83.24) | 5654 (67.56) | <0.01 |
| Yes (BMI≥25 kg/m^2^), n (%) | 29 (16.76) | 2715 (32.44) |  |

^a^ except nonsmokers

SD, standard deviation; BMI, body mass index

Table S2. Association between smoking status, urine cotinine and NNAL concentrations, and incident lung cancer risk: results after multiple imputation.

|  |  | Overall (n=8,542) | | Men (n=3,351) | | Women (n=5,191) | |
| --- | --- | --- | --- | --- | --- | --- | --- |
|  |  | HR (95% C.I.) | P for trend | HR (95% C.I.) | P for trend | HR (95% C.I.) | P for trend |
| Model 1 |  |  |  |  |  |  |  |
| Smoking status | Continuous (log_e_) | **1.02 (1.02, 1.03)** |  | **1.01 (1.01, 1.02)** |  | **1.05 (1.03, 1.07)** |  |
|  | Non-smoker | Reference | <0.001 | Reference | <0.001 | Reference | <0.001 |
|  | <30 pack-years | **4.07 (2.77, 5.99)** |  | **3.03 (1.43, 6.44)** |  | **3.35 (1.77, 6.36)** |  |
|  | ≥30 pack-years | **6.92 (4.85, 9.88)** |  | **4.44 (2.15, 9.19)** |  | **8.87 (3.42, 23.00)** |  |
| Cotinine | Continuous (log_e_) | **1.303 1.246 1.36)** |  | **1.246 (1.173 1.32)** |  | **1.208 (1.102 1.32)** |  |
|  | < 1.41 | Reference | <0.001 | Reference | <0.001 | Reference | 0.002 |
|  | 1.41 - 13.38 | 1.074 (0.674 1.713) |  | 1.04 (0.52, 2.11) |  | 1.11 (0.60, 2.08) |  |
|  | 13.38 - 1082.84 | **3.181 (2.042 4.957)** |  | **2.89 (1.60, 5.22)** |  | 1.41 (0.59, 3.37) |  |
|  | ≥ 1082.84 | **6.556 (4.345 9.893)** |  | **4.71 (2.67, 8.29)** |  | **4.55 (2.10, 9.88)** |  |
| NNAL | Continuous (log_e_) | **1.497 (1.396 1.605)** |  | **1.435 1.307 1.575** |  | **1.294 (1.132 1.479)** |  |
|  | < 3.21 | Reference | <0.001 | Reference | <0.001 | Reference | <0.001 |
|  | 3.21 - 19.30 | 1.05 (0.64, 1.71) |  | 0.85 (0.40, 1.80) |  | 1.27 (0.66, 2.46) |  |
|  | 19.30 - 163.89 | **2.28 (1.43, 3.63)** |  | **2.17 (1.19, 3.95)** |  | 1.24 (0.53, 2.89) |  |
|  | ≥ 163.89 | **7.72 (5.15, 11.58)** |  | **5.29 (3.07, 9.10)** |  | **5.89 (2.83, 12.28)** |  |
| Model 2 |  |  |  |  |  |  |  |
| Smoking status | Continuous (log_e_) | **1.02 (1.01, 1.02)** |  | **1.01 (1.01, 1.02)** |  | **1.05 (1.03, 1.08)** |  |
|  | Non-smoker | Reference | <0.001 | Reference | <0.001 | Reference | <0.001 |
|  | <30 pack-years | **3.08 (1.91, 4.95)** |  | **3.00 (1.40, 6.42)** |  | **2.96 (1.53, 5.73)** |  |
|  | ≥30 pack-years | **4.88 (2.92, 8.15)** |  | **4.42 (2.12, 9.21)** |  | **9.22 (3.43, 24.81)** |  |
| Cotinine | Continuous (log_e_) | **1.21 (1.15, 1.27)** |  | **1.24 (1.16, 1.32)** |  | **1.15 (1.05, 1.27)** |  |
|  | < 1.41 | Reference | <0.001 | Reference | <0.001 | Reference | 0.037 |
|  | 1.41 - 13.38 | 1.03 (0.64, 1.66) |  | 1.04 (0.51, 2.11) |  | 0.93 (0.49, 1.77) |  |
|  | 13.38 - 1082.84 | **2.16 (1.36, 3.43)** |  | **2.80 (1.53, 5.11)** |  | 1.03 (0.42, 2.53) |  |
|  | ≥ 1082.84 | **3.60 (2.29, 5.65)** |  | **4.46 (2.48, 8.04)** |  | **3.00 (1.33, 6.77)** |  |
| NNAL | Continuous (log_e_) | **1.35 (1.25, 1.46)** |  | **1.43 (1.29, 1.57)** |  | **1.23 (1.07, 1.42)** |  |
|  | < 3.21 | Reference | <0.001 | Reference | <0.001 | Reference | 0.005 |
|  | 3.21 - 19.30 | 1.04 (0.63, 1.71) |  | 0.88 (0.41, 1.89) |  | 1.07 (0.53, 2.16) |  |
|  | 19.30 - 163.89 | **1.81 (1.11, 2.93)** |  | **2.22 (1.20, 4.10)** |  | 1.00 (0.41, 2.44) |  |
|  | ≥ 163.89 | **4.75 (3.00, 7.52)** |  | **5.58 (3.12, 9.98)** |  | **4.33 (1.90, 9.90)** |  |

Model 1: unadjusted estimates, Model 2: Adjusted for age, sex, area, enrollment year, alcohol consumption, BMI (kg/m2) and education achievement.

Table S3. Association between urine NNAL concentrations and incident lung cancer risk: results after multiple imputation.

| NNAL | Overall (n=8542) | | Men (n=3351) | | Women (n=5191) | | |
| --- | --- | --- | --- | --- | --- | --- | --- |
|  | HR (95% C.I.) | P for trend | HR (95% C.I.) | P for trend | HR (95% C.I.) | P for trend |  |
| Model 1 |  |  |  |  |  |  |  |
| Continuous (log_e_) | **1.31 (1.21, 1.42)** |  | **1.38 (1.25, 1.53)** |  | 1.11 (0.95, 1.30) |  |  |
| < 3.21 | Reference | <0.001 | Reference | <0.001 | Reference | 0.174 |  |
| 3.21 - 19.30 | 1.02 (0.61, 1.68) |  | 0.86 (0.40, 1.83) |  | 1.05 (0.52, 2.11) |  |  |
| 19.30 - 163.89 | 1.74 (1.07, 2.82) |  | 2.13 (1.16, 3.93) |  | 0.87 (0.35, 2.15) |  |  |
| ≥ 163.89 | 4.00 (2.51, 6.38) |  | 4.73 (2.62, 8.53) |  | 2.51 (0.97, 6.51) |  |  |
| Model 2 |  |  |  |  |  |  |  |
| Continuous (log_e_) | **1.25 (1.08, 1.44)** |  | **1.32 (1.10, 1.58)** |  | 1.13 (0.89, 1.43) |  |  |
| < 3.21 | Reference | 0.007 | Reference | 0.008 | Reference | 0.307 |  |
| 3.21 - 19.30 | 0.98 (0.59, 1.64) |  | 0.82 (0.38, 1.78) |  | 1.10 (0.54, 2.23) |  |  |
| 19.30 - 163.89 | 1.36 (0.70, 2.65) |  | 1.51 (0.62, 3.69) |  | 1.07 (0.37, 3.07) |  |  |
| ≥ 163.89 | **3.07 (1.34, 7.01)** |  | **3.27 (1.14, 9.38)** |  | 5.21 (1.06, 25.71) |  |  |
| Model 3 |  |  |  |  |  |  |  |
| Continuous (log_e_) | **1.22 (1.06, 1.42)** |  | **1.29 (1.08, 1.55)** |  | 1.11 (0.88, 1.41) |  |  |
| < 3.21 | Reference | 0.017 | Reference | 0.018 | Reference | 0.366 |  |
| 3.21 - 19.30 | 0.97 (0.59, 1.62) |  | 0.81 (0.38, 1.74) |  | 1.11 (0.54, 2.27) |  |  |
| 19.30 - 163.89 | 1.38 (0.71, 2.68) |  | 1.53 (0.63, 3.73) |  | 1.10 (0.38, 3.21) |  |  |
| ≥ 163.89 | **2.80 (1.22, 6.41)** |  | **3.00 (1.05, 8.57)** |  | 4.29 (0.83, 22.20) |  |  |

Model 1 is adjusted for age, sex, area, enrollment year, alcohol consumption, BMI (kg/m^2^), education achievement and smoking status (pack-year).

Model 2 is adjusted for age, sex, area, enrollment year, alcohol consumption, BMI (kg/m^2^), education achievement and cotinine level.

Model 3 is adjusted for age, age, sex, area, enrollment year, alcohol consumption, BMI (kg/m^2^), education achievement, smoking status (pack-year) and cotinine level.

Table S4. Association between smoking status, urine cotinine and NNAL concentrations, and incident lung cancer risk (diagnosed after two years of the entry).

|  |  | Overall (n=8,542) | | | Men (n=3,351) | | | Women (n=5,191) | | |
| --- | --- | --- | --- | --- | --- | --- | --- | --- | --- | --- |
|  |  | Cases (%) | HR (95% C.I.) | P for trend | Cases (%) | HR (95% C.I.) | P for trend | Cases (%) | HR (95% C.I.) | P for trend |
| Model 1 |  |  |  |  |  |  |  |  |  |  |
| Smoking status | Continuous (log_e_) |  | **1.02 (1.02, 1.03)** |  |  | **1.02 (1.01, 1.02)** |  |  | **1.05 (1.03, 1.07)** |  |
|  | Non-smoker | 40 (0.74) | Reference | <0.01 | 6 (0.91) | Reference | <0.01 | 34 (0.71) | Reference | <0.01 |
|  | <30 pack-years | 44 (2.57) | **3.88 (2.53, 5.96)** |  | 35 (2.61) | **3.32 (1.40, 7.90)** |  | 9 (2.44) | **2.82 (1.33, 5.97)** |  |
|  | ≥30 pack-years | 70 (5.08) | **6.96 (4.72, 10.27)** |  | 65 (4.87) | **4.89 (2.12, 11.29)** |  | 5 (11.90) | **11.67 (4.46, 30.55)** |  |
| Cotinine | Continuous (log_e_) |  | **1.30 (1.23, 1.36)** |  |  | **1.26 (1.17, 1.35)** |  |  | **1.17 (1.05, 1.30)** |  |
|  | < 1.41 | 26 (0.86) | Reference | <0.01 | 10 (1.25) | Reference | <0.01 | 16 (0.72) | Reference | 0.02 |
|  | 1.41 - 13.38 | 32 (1.06) | 1.20 (0.71, 2.01) |  | 12 (1.49) | 1.22 (0.53, 2.82) |  | 20 (0.90) | 1.20 (0.62, 2.32) |  |
|  | 13.38 - 1082.84 | 37 (2.71) | **3.20 (1.94, 5.28)** |  | 33 (3.95) | **3.41 (1.68, 6.92)** |  | 4 (0.76) | 0.94 (0.31, 2.81) |  |
|  | ≥ 1082.84 | 59 (5.33) | **6.78 (4.27, 10.77)** |  | 51 (5.67) | **5.44 (2.75, 10.78)** |  | 8 (3.85) | **4.17 (1.78, 9.76)** |  |
| NNAL | Continuous (log_e_) |  | **1.52 (1.40, 1.64)** |  |  | **1.51 (1.35, 1.69)** |  |  | **1.25 (1.08, 1.45)** |  |
|  | < 3.21 | 22 (0.74) | Reference | <0.01 | 8 (0.99) | Reference | <0.01 | 14 (0.65) | Reference | <0.01 |
|  | 3.21 - 19.30 | 29 (1.03) | 1.30 (0.75, 2.27) |  | 11 (1.39) | 1.44 (0.58, 3.58) |  | 18 (0.89) | 1.26 (0.63, 2.54) |  |
|  | 19.30 - 163.89 | 30 (1.88) | 2.42 (1.39, 4.19) |  | 24 (2.91) | **3.07 (1.38, 6.84)** |  | 6 (0.78) | 1.03 (0.40, 2.69) |  |
|  | ≥ 163.89 | 73 (6.35) | **8.70 (5.40, 14.03**) |  | 63 (6.91) | **7.78 (3.72, 16.28)** |  | 10 (4.20) | **4.96 (2.19, 11.19)** |  |
| Model 2 |  |  | Reference |  |  | Reference |  |  | Reference |  |
| Smoking status | Continuous (log_e_) |  | **1.02 (1.01, 1.02)** |  |  | **1.02 (1.01, 1.02)** |  |  | **1.06 (1.03, 1.08)** |  |
|  | Non-smoker | 40 (0.74) | Reference | <0.01 | 6 (0.91) | Reference | <0.01 | 34 (0.71) | Reference | <0.01 |
|  | <30 pack-years | 44 (2.57) | **3.14 (1.84, 5.37)** |  | 35 (2.61) | **3.31 (1.38, 7.94)** |  | 9 (2.44) | **2.56 (1.18, 5.58)** |  |
|  | ≥30 pack-years | 70 (5.08) | **5.36 (3.00, 9.56)** |  | 65 (4.87) | **4.83 (2.07, 11.24)** |  | 5 (11.90) | **12.62 (4.54, 35.14)** |  |
| Cotinine | Continuous (log_e_) |  | **1.20 (1.14, 1.27)** |  |  | **1.25 (1.16, 1.35)** |  |  | **1.13 (1.01, 1.26)** |  |
|  | < 1.41 | 26 (0.86) | Reference | <0.01 | 10 (1.25) | Reference | <0.01 | 16 (0.72) | Reference | 0.14 |
|  | 1.41 - 13.38 | 32 (1.06) | 1.13 (0.67, 1.92) |  | 12 (1.49) | 1.20 (0.51, 2.81) |  | 20 (0.90) | 0.97 (0.50, 1.91) |  |
|  | 13.38 - 1082.84 | 37 (2.71) | **2.16 (1.28, 3.66)** |  | 33 (3.95) | **3.23 (1.57, 6.63)** |  | 4 (0.76) | 0.69 (0.22, 2.11) |  |
|  | ≥ 1082.84 | 59 (5.33) | **3.73 (2.23, 6.24)** |  | 51 (5.67) | **5.17 (2.54, 10.52)** |  | 8 (3.85) | **2.92 (1.19, 7.19)** |  |
| NNAL | Continuous (log_e_) |  | **1.38 (1.26, 1.51)** |  |  | **1.48 (1.32, 1.66)** |  |  | **1.23 (1.05, 1.43)** |  |
|  | < 3.21 | 22 (0.74) | Reference | <0.01 | 8 (0.99) | Reference | <0.01 | 14 (0.65) | Reference | 0.02 |
|  | 3.21 - 19.30 | 29 (1.03) | 1.24 (0.70, 2.18) |  | 11 (1.39) | 1.44 (0.57, 3.63) |  | 18 (0.89) | 1.17 (0.56, 2.47) |  |
|  | 19.30 - 163.89 | 30 (1.88) | **1.89 (1.06, 3.35)** |  | 24 (2.91) | **3.11 (1.38, 7.04)** |  | 6 (0.78) | 0.93 (0.34, 2.57) |  |
|  | ≥ 163.89 | 73 (6.35) | **5.31 (3.10, 9.11)** |  | 63 (6.91) | **7.80 (3.58, 16.98)** |  | 10 (4.20) | **4.19 (1.66, 10.55)** |  |

Model 1: unadjusted estimates, Model 2: Adjusted for age, sex, area, enrollment year, alcohol consumption, BMI (kg/m2) and education achievement.

Table S5. Association between urine NNAL concentrations and incident lung cancer risk (diagnosed after two years of the entry).

| NNAL | Overall (n=8,542) | | | Men (n=3,351) | | | Women (n=5,191) | | | |
| --- | --- | --- | --- | --- | --- | --- | --- | --- | --- | --- |
|  | Cases (%) | HR (95% C.I.) | P for trend | Cases (%) | HR (95% C.I.) | P for trend | Cases (%) | HR (95% C.I.) | P for trend |  |
| Model 1 |  |  |  |  |  |  |  |  |  |  |
| Continuous (log_e_) |  | **1.33 (1.21, 1.45)** |  |  | **1.43 (1.27, 1.61)** |  |  | 1.11 (0.94, 1.31) |  |  |
| < 3.21 | 22 (0.74) | Reference | <0.001 | 8 (0.99) | Reference | <0.001 | 14 (0.65) | Reference | 0.310 |  |
| 3.21 - 19.30 | 29 (1.03) | 1.22 (0.69, 2.15) |  | 11 (1.39) | 1.42 (0.57, 3.59) |  | 18 (0.89) | 1.14 (0.54, 2.41) |  |  |
| 19.30 - 163.89 | 30 (1.88) | **1.82 (1.03, 3.23)** |  | 24 (2.91) | **3.01 (1.33, 6.82)** |  | 6 (0.78) | 0.80 (0.29, 2.22) |  |  |
| ≥ 163.89 | 73 (6.35) | **4.38 (2.53, 7.57)** |  | 63 (6.91) | **6.49 (2.95, 14.25)** |  | 10 (4.20) | 2.30 (0.81, 6.56) |  |  |
| Model 2 |  |  |  |  |  |  |  |  |  |  |
| Continuous (log_e_) |  | **1.32 (1.13, 1.55)** |  |  | **1.41 (1.16, 1.72)** |  |  | 1.21 (0.94, 1.56) |  |  |
| < 3.21 | 22 (0.74) | Reference | 0.003 | 8 (0.99) | Reference | 0.003 | 14 (0.65) | Reference | 0.241 |  |
| 3.21 - 19.30 | 29 (1.03) | 1.20 (0.68, 2.13) |  | 11 (1.39) | 1.37 (0.54, 3.48) |  | 18 (0.89) | 1.23 (0.58, 2.63) |  |  |
| 19.30 - 163.89 | 30 (1.88) | 1.58 (0.75, 3.32) |  | 24 (2.91) | 2.32 (0.80, 6.78) |  | 6 (0.78) | 1.14 (0.36, 3.59) |  |  |
| ≥ 163.89 | 73 (6.35) | **3.98 (1.58, 10.03**) |  | 63 (6.91) | **5.16 (1.50, 17.76)** |  | 10 (4.20) | **7.04 (1.27, 39.18)** |  |  |
| Model 3 |  |  |  |  |  |  |  |  |  |  |
| Continuous (log_e_) |  | **1.30 (1.11, 1.53)** |  |  | **1.39 (1.14, 1.69)** |  |  | 1.18 (0.92, 1.52) |  |  |
| < 3.21 | 22 (0.74) | Reference | 0.007 | 8 (0.99) | Reference | 0.006 | 14 (0.65) | Reference | 0.299 |  |
| 3.21 - 19.30 | 29 (1.03) | 1.19 (0.67, 2.12) |  | 11 (1.39) | 1.37 (0.54, 3.48) |  | 18 (0.89) | 1.24 (0.58, 2.67) |  |  |
| 19.30 - 163.89 | 30 (1.88) | 1.62 (0.77, 3.40) |  | 24 (2.91) | 2.42 (0.83, 7.03) |  | 6 (0.78) | 1.16 (0.36, 3.76) |  |  |
| ≥ 163.89 | 73 (6.35) | **3.63 (1.44, 9.16)** |  | 63 (6.91) | **4.77 (1.39, 16.36)** |  | 10 (4.20) | 5.51 (0.93, 32.80) |  |  |

Model 1 is adjusted for age, sex, area, enrollment year, alcohol consumption, BMI (kg/m^2^), education achievement and smoking status (pack-year).

Model 2 is adjusted for age, sex, area, enrollment year, alcohol consumption, BMI (kg/m^2^), education achievement and cotinine level.

Model 3 is adjusted for age, age, sex, area, enrollment year, alcohol consumption, BMI (kg/m^2^), education achievement, smoking status (pack-year) and cotinine level.

Table S6. Association between smoking status, urine cotinine and NNAL concentrations, and incident lung cancer risk (diagnosed after five years of the entry).

|  |  | Overall (n=8,542) | | | Men (n=3,351) | | | Women (n=5,191) | | |
| --- | --- | --- | --- | --- | --- | --- | --- | --- | --- | --- |
|  |  | Cases (%) | HR (95% C.I.) | P for trend | Cases (%) | HR (95% C.I.) | P for trend | Cases (%) | HR (95% C.I.) | P for trend |
| Model 1 |  |  |  |  |  |  |  |  |  |  |
| Smoking status | Continuous (log_e_) |  | **1.02 (1.02, 1.03)** |  |  | **1.01 (1.00, 1.02)** |  |  | **1.05 (1.03, 1.08)** |  |
|  | Non-smoker | 34 (0.63) | Reference | <.0001 | 5 (0.76) | Reference | 0.002 | 29 (0.61) | Reference | <0.001 |
|  | <30 pack-years | 32 (1.88) | 3.29 (2.03, 5.34) |  | 24 (1.80) | **2.74 (1.05, 7.19)** |  | 8 (2.17) | **2.88 (1.30, 6.41)** |  |
|  | ≥30 pack-years | 48 (3.54) | **5.60 (3.61, 8.70)** |  | 43 (3.27) | **3.85 (1.52, 9.73)** |  | 5 (11.90) | **13.27 (5.00, 35.23)** |  |
| Cotinine | Continuous (log_e_) |  | **1.30 (1.23, 1.38)** |  |  | **1.31 (1.19, 1.43)** |  |  | **1.19 (1.07, 1.32)** |  |
|  | < 1.41 | 20 (0.66) | Reference | <.0001 | 7 (0.88) | Reference | <0.001 | 13 (0.59) | Reference | 0.013 |
|  | 1.41 - 13.38 | 24 (0.80) | 1.19 (0.66, 2.15) |  | 6 (0.75) | 0.90 (0.30, 2.68) |  | 18 (0.81) | 1.33 (0.65, 2.73) |  |
|  | 13.38 - 1082.84 | 27 (1.99) | **3.08 (1.73, 5.49)** |  | 23 (2.79) | **3.51 (1.50, 8.18)** |  | 4 (0.76) | 1.15 (0.37, 3.52) |  |
|  | ≥ 1082.84 | 43 (3.94) | **6.60 (3.88, 11.24)** |  | 36 (4.07) | **5.91 (2.61, 13.37)** |  | 7 (3.38) | **4.42 (1.76, 11.12)** |  |
| NNAL | Continuous (log_e_) |  | **1.51 (1.38, 1.65)** |  |  | **1.56 (1.36, 1.78)** |  |  | **1.29 (1.10, 1.50)** |  |
|  | < 3.21 | 17 (0.57) | Reference | <0.001 | 5 (0.62) | Reference | <0.001 | 12 (0.56) | Reference | 0.004 |
|  | 3.21 - 19.30 | 21 (0.75) | 1.24 (0.65, 2.35) |  | 6 (0.76) | 1.30 (0.40, 4.27) |  | 15 (0.75) | 1.23 (0.58, 2.63) |  |
|  | 19.30 - 163.89 | 22 (1.39) | **2.33 (1.24, 4.39)** |  | 16 (1.96) | **3.37 (1.23, 9.19)** |  | 6 (0.78) | 1.20 (0.45, 3.20) |  |
|  | ≥ 163.89 | 54 (4.77) | **8.53 (4.94, 14.72)** |  | 45 (5.03) | **9.47 (3.75, 23.93)** |  | 9 (3.80) | **5.14 (2.16, 12.25**) |  |
| Model 2 |  |  |  |  |  |  |  |  |  |  |
| Smoking status | Continuous (log_e_) |  | **1.02 (1.01, 1.02)** |  |  | **1.01 (1.00, 1.02)** |  |  | **1.06 (1.03, 1.08)** |  |
|  | Non-smoker | 34 (0.63) | Reference | <0.001 | 5 (0.76) | Reference | 0.005 | 29 (0.61) | Reference | <0.001 |
|  | <30 pack-years | 32 (1.88) | 2.91 (1.61, 5.27) |  | 24 (1.80) | **2.71 (1.02, 7.21)** |  | 8 (2.17) | **2.58 (1.12, 5.91)** |  |
|  | ≥30 pack-years | 48 (3.54) | **4.85 (2.52, 9.33)** |  | 43 (3.27) | **3.65 (1.43, 9.36)** |  | 5 (11.90) | **14.09 (4.95, 40.09)** |  |
| Cotinine | Continuous (log_e_) |  | **1.21 (1.13, 1.29)** |  |  | **1.27 (1.16, 1.40)** |  |  | **1.14 (1.01, 1.28)** |  |
|  | < 1.41 | 20 (0.66) | Reference | <0.001 | 7 (0.88) | Reference | <0.001 | 13 (0.59) | Reference | 0.130 |
|  | 1.41 - 13.38 | 24 (0.80) | 1.00 (0.55, 1.83) |  | 6 (0.75) | 0.76 (0.25, 2.30) |  | 18 (0.81) | 1.07 (0.52, 2.24) |  |
|  | 13.38 - 1082.84 | 27 (1.99) | **1.98 (1.08, 3.63)** |  | 23 (2.79) | **2.83 (1.20, 6.70)** |  | 4 (0.76) | 0.81 (0.26, 2.57) |  |
|  | ≥ 1082.84 | 43 (3.94) | **3.37 (1.86, 6.08)** |  | 36 (4.07) | **4.44 (1.90, 10.37)** |  | 7 (3.38) | **2.95 (1.11, 7.80)** |  |
| NNAL | Continuous (log_e_) |  | **1.36 (1.22, 1.51)** |  |  | **1.48 (1.28, 1.71)** |  |  | **1.25 (1.06, 1.47)** |  |
|  | < 3.21 | 17 (0.57) | Reference | <0.001 | 5 (0.62) | Reference | <0.001 | 12 (0.56) | Reference | 0.021 |
|  | 3.21 - 19.30 | 21 (0.75) | 1.01 (0.52, 1.94) |  | 6 (0.76) | 1.10 (0.33, 3.68) |  | 15 (0.75) | 1.08 (0.48, 2.43) |  |
|  | 19.30 - 163.89 | 22 (1.39) | 1.64 (0.85, 3.17) |  | 16 (1.96) | **2.80 (1.00, 7.83)** |  | 6 (0.78) | 1.04 (0.37, 2.93) |  |
|  | ≥ 163.89 | 54 (4.77) | **4.66 (2.50, 8.68)** |  | 45 (5.03) | **7.14 (2.68, 19.06)** |  | 9 (3.80) | **4.16 (1.56, 11.10)** |  |

Model 1: unadjusted estimates, Model 2: Adjusted for age, sex, area, enrollment year, alcohol consumption, BMI (kg/m2) and education achievement.

Table S7. Association between urine NNAL concentrations and incident lung cancer risk (diagnosed after five years of the entry).

| NNAL | Overall (n=8,542) | | | Men (n=3,351) | | | Women (n=5,191) | | | |
| --- | --- | --- | --- | --- | --- | --- | --- | --- | --- | --- |
|  | Cases (%) | HR (95% C.I.) | P for trend | Cases (%) | HR (95% C.I.) | P for trend | Cases (%) | HR (95% C.I.) | P for trend |  |
| Model 1 |  |  |  |  |  |  |  |  |  |  |
| Continuous (log_e_) |  | **1.32 (1.18, 1.47)** |  |  | **1.45 (1.25, 1.68)** |  |  | **1.12 (0.94, 1.34)** |  |  |
| < 3.21 | 17 (0.57) | Reference | <0.001 | 5 (0.62) | Reference | <0.001 | 12 (0.56) | Reference | 0.317 |  |
| 3.21 - 19.30 | 21 (0.75) | 1.00 (0.52, 1.93) |  | 6 (0.76) | 1.11 (0.33, 3.69) |  | 15 (0.75) | 1.05 (0.47, 2.37) |  |  |
| 19.30 - 163.89 | 22 (1.39) | 1.61 (0.83, 3.13) |  | 16 (1.96) | **2.79 (1.00, 7.79)** |  | 6 (0.78) | 0.88 (0.31, 2.51) |  |  |
| ≥ 163.89 | 54 (4.77) | **4.08 (2.16, 7.71)** |  | 45 (5.03) | **6.58 (2.43, 17.81)** |  | 9 (3.80) | 2.22 (0.73, 6.75) |  |  |
| Model 2 |  |  |  |  |  |  |  |  |  |  |
| Continuous (log_e_) |  | **1.24 (1.02, 1.50)** |  |  | 1.28 (0.99, 1.66) |  |  | 1.24 (0.94, 1.62) |  |  |
| < 3.21 | 17 (0.57) | Reference | 0.038 | 5 (0.62) | Reference | 0.043 | 12 (0.56) | Reference | 0.299 |  |
| 3.21 - 19.30 | 21 (0.75) | 0.96 (0.50, 1.87) |  | 6 (0.76) | 1.01 (0.30, 3.41) |  | 15 (0.75) | 1.13 (0.50, 2.56) |  |  |
| 19.30 - 163.89 | 22 (1.39) | 1.31 (0.56, 3.06) |  | 16 (1.96) | 1.72 (0.45, 6.52) |  | 6 (0.78) | 1.21 (0.36, 4.04) |  |  |
| ≥ 163.89 | 54 (4.77) | **3.18 (1.08, 9.32)** |  | 45 (5.03) | 3.55 (0.77, 16.46) |  | 9 (3.80) | 6.17 (0.97, 39.27) |  |  |
| Model 3 |  |  |  |  |  |  |  |  |  |  |
| Continuous (log_e_) |  | **1.22 (1.01, 1.48)** |  |  | 1.27 (0.97, 1.65) |  |  | 1.20 (0.91, 1.58) |  |  |
| < 3.21 | 17 (0.57) | Reference | 0.054 | 5 (0.62) | Reference | 0.054 | 12 (0.56) | Reference | 0.370 |  |
| 3.21 - 19.30 | 21 (0.75) | 0.97 (0.50, 1.88) |  | 6 (0.76) | 1.02 (0.30, 3.45) |  | 15 (0.75) | 1.13 (0.50, 2.59) |  |  |
| 19.30 - 163.89 | 22 (1.39) | 1.34 (0.57, 3.14) |  | 16 (1.96) | 1.77 (0.47, 6.73) |  | 6 (0.78) | 1.23 (0.36, 4.24) |  |  |
| ≥ 163.89 | 54 (4.77) | **3.00 (1.02, 8.81)** |  | 45 (5.03) | 3.48 (0.75, 16.09) |  | 9 (3.80) | 4.79 (0.70, 32.65) |  |  |

Model 1 is adjusted for age, sex, area, enrollment year, alcohol consumption, BMI (kg/m^2^), education achievement and smoking status (pack-year).

Model 2 is adjusted for age, sex, area, enrollment year, alcohol consumption, BMI (kg/m^2^), education achievement and cotinine level.

Model 3 is adjusted for age, age, sex, area, enrollment year, alcohol consumption, BMI (kg/m^2^), education achievement, smoking status (pack-year) and cotinine level.


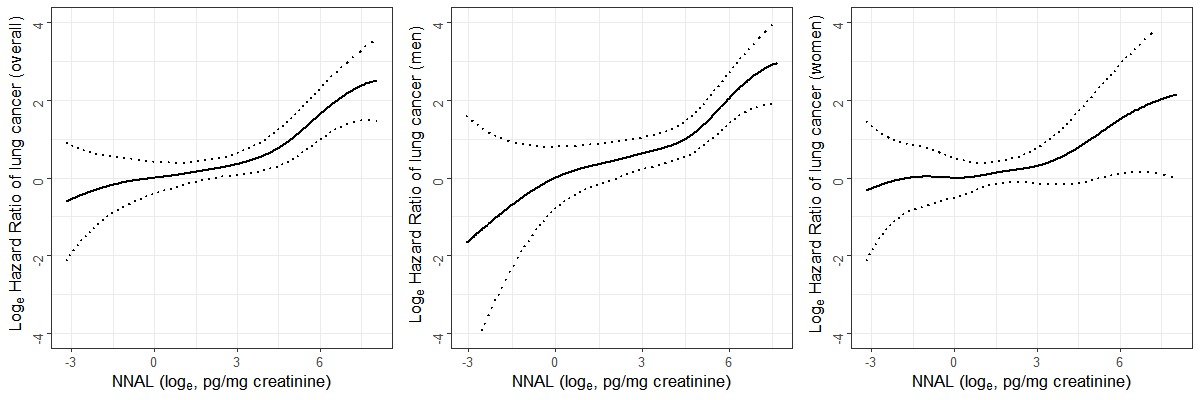


**Figure S1. Association between urine NNAL concentrations and incidental lung cancer risk**

A restricted cubic spline model was used for urinary NNAL to allow for nonlinear associations. Hazard ratios were obtained using a Cox proportional hazard analysis, adjusting for age, sex, area, enrollment year, alcohol consumption, BMI (kg/m^2^), education achievement, smoking status (pack-years) and cotinine concentration. A urine NNAL level of 1 pg/g creatinine was set as the reference. Hazard ratios are given for 1-unit increase in natural log_e_–transformed urine NNAL value. The dashed lines indicate 95% CIs.
